# Infliximab-associated multifocal central nervous system demyelination mimicking multiple sclerosis in a patient with Crohn’s disease: a case report

**DOI:** 10.1186/s12883-026-04932-3

**Published:** 2026-04-30

**Authors:** Mohammad Ahmad Sawaftah, Mohammed AbuBaha, Bara AbuBaha, Muthanna Shoman, Mohammad muneeb izzat Abbas

**Affiliations:** 1 Internal Medicine Department, Al-Watani Governmental Hospital, Nablus, Palestine; 2 Department of Medicine, An-Najah National University, Nablus, Palestine

**Keywords:** Infliximab, Anti-TNF-α, Crohn’s disease, Central nervous system demyelination, Multiple sclerosis mimic, Biologic therapy adverse event, T2/FLAIR MRI lesions, Oligoclonal bands

## Abstract

**Background:**

Tumor necrosis factor-alpha (TNF-α) inhibitors are widely used for immune-mediated inflammatory diseases, including Crohn’s disease. Although generally effective, TNF-α blockade has been linked to uncommon but clinically significant paradoxical immune-mediated neurologic adverse events, including inflammatory demyelinating disorders of the central nervous system (CNS).

**Case presentation:**

A 32-year-old woman with Crohn’s disease (perianal fistula) treated with infliximab for one year presented with progressive dizziness, headache, and bilateral lower-limb weakness. Neurologic examination demonstrated ataxic gait, intention tremor, dysarthria, brisk reflexes (right-predominant), and a positive right Babinski sign, with preserved sensation. Initial laboratory testing and head CT were unremarkable. Brain MRI revealed multiple multifocal T2/FLAIR hyperintense lesions involving bilateral periventricular and deep white matter, with plaques affecting the thalami, internal capsule, corpus callosum, cerebellum, and cerebellar peduncles; spinal MRI showed no active lesions. Cerebrospinal fluid studies were normal; infectious testing and autoimmune screening were negative, and oligoclonal bands and serum aquaporin-4 antibodies were negative. Infliximab was discontinued, and high-dose pulse corticosteroids were administered, resulting in mild symptomatic improvement; however, ataxia and dysarthria persisted. At three months, follow-up MRI showed stable lesions without radiologic activity alongside slow clinical improvement with rehabilitation.

**Conclusion:**

This case highlights a clinically significant, infliximab-associated CNS demyelinating syndrome with an MS-like radiologic pattern in a patient without prior neurologic history. Prompt recognition, discontinuation of the suspected agent, and early immunotherapy are essential; nonetheless, incomplete recovery may occur, underscoring the need for careful neurologic vigilance in patients receiving anti-TNF therapy.

## Introduction

Tumor necrosis factor-alpha (TNF-α) are biologic agent known to have an important emerging role in the treatment of immune-mediated inflammatory diseases, such as Crohn’s disease, rheumatoid arthritis, and spondyloarthritis, responded better with these agents. In Refractory patients, these biologic agents have significantly improved disease control and reduced possible complications of the disease [1]. Infliximab, a chimeric monoclonal antibody, goes after TNF-α directly and is particularly effective in people with moderate to severe Crohn’s disease or those struggling with fistulas. However, infliximab has been associated with rare but potentially serious paradoxical immune-mediated adverse events [2].

TNF-α has a dual role in immune regulation and central nervous system (CNS) homeostasis. TNF-α drives chronic inflammation, but it also contributes to the protection of neurons, supports remyelination, and participates in immune surveillance within the CNS [3]. TNF-α inhibitors may disrupt this balance, and because of that, some people may be at risk for inflammatory demyelinating disorders, including MS-like disease, optic neuritis, transverse myelitis, and acute disseminated encephalomyelitis [2]. These conditions often mimic idiopathic demyelinating diseases, which makes both diagnosis and treatment much trickier.

Among TNF-α inhibitors, infliximab stands out as one of the most often reported in cases of biologic-associated CNS demyelination [4]. Several case reports and observational studies show that patients sometimes develop new demyelinating syndromes months or even years after starting infliximab, even patients with no previous neurological problems may develop new demyelinating syndromes [5]. Radiologically, these cases usually show multifocal T2/FLAIR hyperintense lesions scattered through the periventricular, juxtacortical, cerebellar, and brainstem regions, giving a feature similar to MS [6]. Patients often get better, or disease stabilization occurs once they stop infliximab and start corticosteroids, supporting a causal relationship [4–6].

Despite growing recognition, TNF-α inhibitor–associated demyelination remains uncommon, and its true incidence may still be underestimated. Large registry studies point to a low absolute risk, but the potential severity of neurological involvement necessitates early recognition and prompt management [1]. Recent recommendations advise the avoidance of TNF-α inhibitors in anyone with a personal or strong family history of demyelinating disease. They also emphasized the need for close neurological monitoring throughout treatment [7].

This case stands out for many reasons. Severe CNS demyelination symptoms appear after long-term infliximab treatment for Crohn’s disease, but the patient has a clean neurological history without any family predisposition. Also, lesions spread across multiple areas mimicking a primary multiple sclerosis appeared with MRI. Cerebrospinal fluid (CSF) analysis showed negative oligoclonal bands, which reduced support for a diagnosis of typical multiple sclerosis in this clinical context. Serum aquaporin-4 antibody negativity was instead helpful in arguing against neuromyelitis optica spectrum disorder rather than multiple sclerosis. Finally, the patient’s neurological recovery was incomplete even after stopping infliximab and starting steroids, this should rise the suspicions for persistent morbidity even after early intervention. Cases like this highlight how unpredictable infliximab-induced CNS demyelination can be.

## Case presentation

A 32-year-old woman with known history of Crohn’s disease complicated by a perianal fistula, presented complaining from worsening neurological symptoms of two months duration. Her diagnosis with Crohn’s diagnosis was confirmed by a biopsy of a perianal ulcer in 2021. She had been treated with azathioprine and corticosteroids, after that transitioned to infliximab therapy administered every eight weeks. She had no prior personal or family history of neurological disease.

Two months before admission, the patient experienced a gradual onset of bilateral proximal lower-limb weakness, it began with difficulty rising from a seated position and climbing stairs, which later progressed to impaired ambulation. With these symptoms, dizziness and bifrontal tension-type headache present. Nausea, vomiting, photophobia, or fever denied by the patient. Family noticed progressive gait instability and slowness of speech. There was no history of sensory loss, bowel or bladder dysfunction, visual loss, diplopia, seizures, loss of consciousness, recent infection, vaccination, trauma, or constitutional symptoms.

The patient was conscious, alert, and oriented to time, place, and person at the time of presentation. Neurological exam performed, wide-based ataxic gait, dysarthria, and intention tremor appeared in the exam. Motor examination looked normal with normal tone and power and no proximal muscle weakness appeared in the exam, reflexes were brisk especially on the right side, and there was a positive right Babinski sign. Sensation was normal. Cranial nerves were intact, no focal deficits. No signs of meningeal irritation. The rest of the systemic exam didn’t show anything unusual.

Initial laboratory tests including CBC, kidney and liver panels, electrolytes, and arterial blood gas, all were normal. Autoimmune screening including antinuclear antibodies (ANA), anti-Ro/SSA, anti-La/SSB, anti-proteinase-3 antibodies, and rheumatoid factor, all were negative. Endocrine evaluation showed that she had panhypopituitarism, with low follicle-stimulating hormone (FSH) and luteinizing hormone (LH), high prolactin, and low vitamin D. Thyroid tests confirmed she was hypothyroid, consistent with her known medical history.

**Fig. 1:**
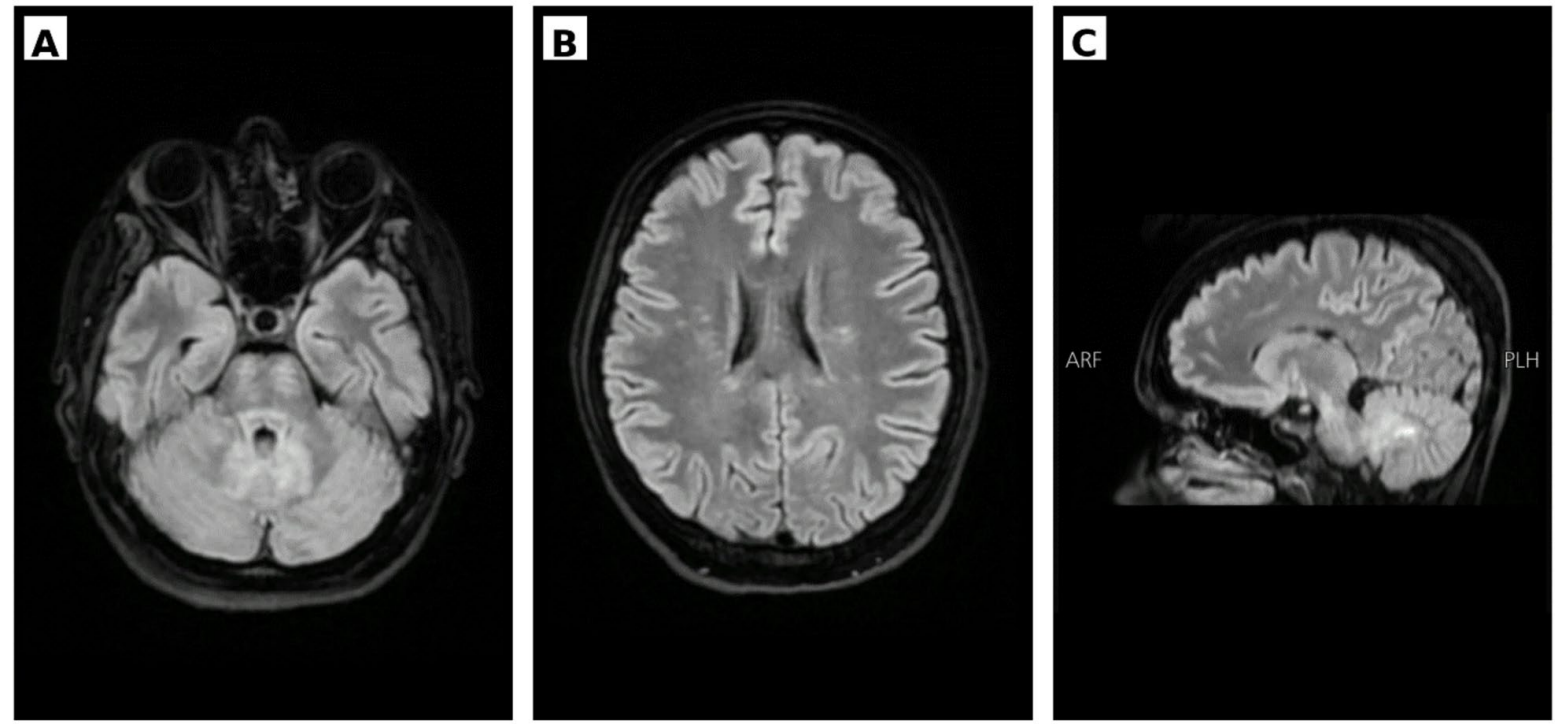
**A** (Axial T2 FLAIR – infratentorial level): Axial T2 fluid-attenuated inversion recovery (FLAIR) image at the level of the brainstem demonstrating hyperintense demyelinating lesions involving the pons and cerebellar white matter, consistent with infratentorial involvement. **B** (Axial T2 FLAIR – supratentorial level): Axial T2 fluid-attenuated inversion recovery (FLAIR) showing multiple bilateral hyperintense lesions in the periventricular and subcortical white matter, in keeping with an demyelinating process. **C** (Sagittal T2 FLAIR): Sagittal T2 fluid-attenuated inversion recovery (FLAIR) image demonstrating hyperintense white-matter lesions involving the periventricular region and posterior fossa, supporting the diagnosis of central nervous system demyelination

Subsequently, A Brain MRI revealed multiple hyperintense T2/FLAIR WI signal intensity lesions in bilateral periventricular and deep white matter, with some plaques involving thalami, internal capsules, corpus callosum, cerebellum, cerebellar peduncles, brainstem, and dentate nuclei, with a predominantly symmetrical distribution. Whole spine MRI did not show spinal cord lesions. Gadolinium enhancement status was not demonstrated on the available figure set and therefore active demyelination could not be established radiologically from the included images alone. No black holes, optic nerve involvement, central vein sign, or paramagnetic rim lesions were identified on the available MRI studies. These findings were suggestive of an inflammatory demyelinating process with a broad differential diagnosis, including multiple sclerosis. A 12-lead electrocardiogram (EKG) did not show any cardiac arrhythmias. The transthoracic echocardiogram failed to show any abnormalities (Fig. 1).

A lumbar puncture was subsequently performed with normal cerebrospinal fluid studies, revealed normal opening pressure, cell count, protein, and glucose levels. Routine CSF analysis showed no pleocytosis or biochemical evidence of infection. Formal CSF microbiological details, including tuberculosis-directed testing, were not available for inclusion. Oligoclonal bands were absent. Serum aquaporin-4 antibodies and myelin oligodendrocyte glycoprotein (MOG) antibodies were also negative, arguing against Neuromyelitis Optica Spectrum Disorder (NMOSD) and MOG-associated disease. CT scans of the chest, abdomen, and pelvis did not show any findings consistent with malignancy or lymphadenopathy.

Based on the clinical presentation, radiologic findings, and exclusion of alternative etiologies. Infliximab was stopped, and a diagnosis of infliximab-associated central nervous system (CNS) demyelinating disease was considered. The patient was started on pulse steroids during her hospital stay, resulting in mild improvement in her weakness and dizziness. However, she continued to have ataxia and dysarthria. After 3 months of neurology follow-up and rehabilitation, she exhibited slow improvement in her symptoms. A follow-up MRI was performed, revealing that the previous demyelinating lesions remained stable with no evidence of disease activity. Follow-up MRI confirmed radiologic stability at 3 months; however, representative follow-up images were not available for inclusion.

## Discussion

Tumor necrosis factor alpha (TNF-α) inhibitors are widely used in the management of inflammatory bowel disease (IBD) due to their efficacy in reducing immune-mediated inflammation. Despite their therapeutic benefit, TNF-α inhibitors have been associated with rare but significant neurological adverse effects, including central and peripheral nervous system demyelination. Although TNF-α blockade was shown to suppress experimental autoimmune encephalomyelitis (EAE), an animal model of multiple sclerosis (MS), clinical trials in humans paradoxically demonstrated worsening of MS activity, leading to contraindication of TNF-α inhibitors in patients with known demyelinating disease [8, 9].

The relationship between TNF-α signaling and MS pathogenesis is complex and incompletely understood. Genetic studies have identified the rs1800693 polymorphism in the TNFRSF1A gene, encoding TNF receptor 1, as a causal variant associated with MS susceptibility. This variant leads to increased production of a soluble TNF receptor that functionally inhibits TNF-α signaling. It has been hypothesized that pharmacologic TNF-α inhibition may mimic this genetic mechanism, thereby increasing MS risk; however, direct causal evidence remains limited [10].

Several mechanisms have been proposed to explain TNF-α inhibitor–associated demyelination. These include alteration of cytokine balance with upregulation of pro‑inflammatory cytokines such as interferon‑γ, loss of regulatory control over autoreactive T lymphocytes, and molecular mimicry or immune complex formation that exposes central nervous system antigens. Through these mechanisms, TNF‑α inhibition may trigger or unmask autoimmune demyelinating processes [7].

In this report, we describe a case of CNS demyelination in a patient with Crohn’s disease treated with infliximab, a chimeric monoclonal anti‑TNF‑α antibody. The temporal relationship between infliximab initiation and neurological symptom onset, in the absence of prior infection, vaccination, or demyelinating disease, suggests a treatment‑related adverse effect. Histopathological, radiographic, and clinical findings raise the question of whether infliximab induces a distinct demyelinating entity or precipitates MS in genetically susceptible individuals [11]. **(**Table 1**)** The main question in this case report is does this patient actually met the formal criteria for multiple sclerosis. The brain MRI showed lesions in typical MS locations, but the patient only had one demyelinating episode. No lesions were detected on the spinal MRI, and there were no oligoclonal bands in the cerebrospinal fluid. In addition, the available images did not demonstrate gadolinium enhancement, so dissemination in time could not be established radiologically from the included studies. However, an alternative explanation namely “infliximab-associated CNS demyelination” was more likely in this clinical context because of the known relationship with anti-TNF exposure and the exclusion of other major etiologies. Based on available date, the patient didn’t meet the 2017 McDonald criteria for definite MS. Also, the available findings were still insufficient to confidently establish a diagnosis of definite MS using the 2024 revision especially without biomarkers or dissemination criteria beyond this one event. Therefore, the case matched infliximab-associated CNS demyelination with an MS-like radiologic pattern, not idiopathic MS.

**Table 1:** Differential Diagnosis of CNS Demyelination and Exclusion in the Present Case

Differential Diagnosis	Typical Features	Findings in Present Case	Reason for Exclusion
Multiple sclerosis (MS)	OCB positive, periventricular lesions, and dissemination in time and space	MS-like MRI pattern; OCB negative; no prior attacks	Did not clearly fulfill 2017 McDonald criteria; available data were also insufficient to confirm definite MS under the 2024 revision
Neuromyelitis optica spectrum disorder (NMOSD)	AQP4 positive, longitudinal spinal lesions, and optic neuritis	No optic neuritis; spinal MRI normal; AQP4 negative	Serology and imaging negative
Myelin oligodendrocyte glycoprotein antibody-associated disease (MOGAD)	MOG antibody positive, optic neuritis, longitudinally extensive myelitis, or bilateral/fluffy poorly demarcated brain lesions	No optic neuritis; no longitudinally extensive myelitis; MOG antibody negative	Serology negative
Acute disseminated encephalomyelitis (ADEM)	Encephalopathy, monophasic illness	No encephalopathy	Clinical presentation inconsistent
Infectious demyelination	Pleocytosis in the CSF and systemic infection	CSF normal; viral panel negative	Infection ruled out
CNS vasculitis	Autoimmune indicators, headache, and ischemic or inflammatory CNS lesions	Vasculitis panel negative	Laboratory and imaging negative
Paraneoplastic syndrome	Loss of weight and cancer on imaging	CT chest/abdomen/pelvis normal	No evidence of malignancy
Drug-induced demyelination	Temporal correlation with drug exposure	Symptoms after 1 year of infliximab	Most consistent diagnosis

Based on clinical features and diagnostic criteria, peripheral demyelinating disorders such as Chronic Inflammatory Demyelinating Polyradiculoneuropathy (CIDP) were considered less likely because the patient had brisk reflexes and a Babinski sign, supporting a central rather than peripheral demyelinating process. In the present case, the deterioration of neurological symptoms following anti‑TNF‑α exposure supported a secondary immune‑mediated process [12].

Currently, there are no standardized treatment guidelines for TNF‑α inhibitor–induced demyelination. Management strategies are largely extrapolated from MS relapse protocols and published case reports. Immediate discontinuation of the offending TNF‑α inhibitor is the cornerstone of treatment and may result in symptom stabilization or improvement. High‑dose corticosteroids are frequently administered and have been shown to accelerate neurological recovery [8].

In patients with persistent or severe disease, additional therapies such as intravenous immunoglobulin (IVIG) or plasma exchange may be considered. IVIG is typically administered as a loading dose of 0.4 g/kg/day for 2–5 days, followed by maintenance dosing of 1 g/kg every three weeks. Plasma exchange may be reserved for refractory cases. Early initiation of therapy is crucial to prevent irreversible neurological damage [2].

Multiple anti‑TNF‑α agents, including infliximab, adalimumab, etanercept, golimumab, and certolizumab pegol, have been implicated in demyelinating disorders of the nervous system. Meta‑analyses have estimated a 36–38% increased risk of inflammatory CNS disease associated with TNF‑α inhibitor therapy. Although prognosis is generally favorable with prompt recognition and intervention, disease severity and outcomes remain variable [13].

Although TNF‑α inhibitor–induced CNS demyelination is rare in pediatric populations, clinicians should remain vigilant due to the potential for a fulminant clinical course. With the expanding use of biologic therapies, further case accumulation is anticipated and will be essential in clarifying disease mechanisms, risk stratification, and optimal management strategies. Early recognition and intervention remain critical to improving neurological outcomes [6].

## Conclusion

Infliximab may be linked to uncommon but clinically important central nervous system inflammatory demyelinating events that resemble multiple sclerosis both clinically and radiologically. When patients on anti-TNF medication have new neurologic symptoms (such as weakness, dysarthria, ataxia, or dizziness), early neurologic evaluation with MRI and ruling out other causes is crucial. Although residual impairments may continue, promptly stopping the suspected drug and starting immunotherapy (usually high-dose corticosteroids) may stabilize disease activity and promote recovery. When providing anti-TNF medications for Crohn’s disease, this instance emphasizes the need for continuous neurologic monitoring and customized risk-benefit reevaluation.

## Data Availability

Data will be provided on request from the editor-in-chief due to limitations.

## Abbreviations

CNS: Central nervous system
TNF-α: Tumor necrosis factor alpha
anti-TNF: Anti–tumor necrosis factor (therapy)
MRI: Magnetic resonance imaging
CT: Computed tomography
CSF: Cerebrospinal fluid
FLAIR: Fluid-attenuated inversion recovery
AQP4: Aquaporin-4 (antibody)
